# Supplementary material for: Expression of Interferons Lambda 3 and 4 Induces Identical Response in Human Liver Cell Lines Depending Exclusively on Canonical Signaling
Source: Int J Mol Sci. 2021 Mar 4;22(5):2560. doi: 10.3390/ijms22052560 (PMC7961969; doi:10.3390/ijms22052560)
Supplement: Supplementary file 1 [file ijms-22-02560-s001.zip › Supplemental data.pdf]

Supplementary material to

Interferons lambda 3 and 4 induce identical response in human liver cell lines depending exclusively on canonical signaling

Mariia Lunova[1*], Jan Kubovciak[2], Barbora Smolková[3], Mariia Uzhytchak[3], Kyra Michalova[4], Alexandr Dejneka[3], Pavel Strnad[5], Oleg Lunov[3], Milan Jirsa[1,4]

[1]Institute for Clinical and Experimental Medicine, 140 21 Prague, Czech Republic
[2]Institute of Molecular Genetics of the Czech Academy of Sciences, Prague, Czech Republic
[3]Institute of Physics of the Czech Academy of Sciences, Prague, Czech Republic
[4]Institute of Medical Biochemistry and Laboratory Diagnostics, General University Hospital and 1st Faculty of Medicine of Charles University, Prague, Czech Republic
[5]Department of Internal Medicine III, University Hospital RWTH (Rheinisch-Westfälisch Technische Hochschule) Aachen, Aachen, Germany

[*]Correspondence: lunm@ikem.cz

Supplementary figure legends

Supplementary Figure 1: **Expression of IFNLs and ISGs in HuH7control cells transfected by IFNL3 and IFNL4 lacking HaloTag.** (A) High resolution confocal image of IFNL3 and IFNL4 in wild type HuH7 transfected with tagged and non-tagged *IFNL3* or *IFNL4* for 48 hours. Empty plasmid was used as a Mock control. Blue – nuclei, green - IFNL3 / IFNL4. IFNL3 and IFNL4 were detected either with the corresponding anti-IFNL3/anti-IFNL4 antibody or with anti-Halo tag ligand. Scale bars 30 µm. (B) Relative expression of *RSAD*, *IFI27*, *IFITM1* and *OAS1* determined in HuH7 cells 48 hours transfected with tagged or non-tagged IFNL3 or IFNL4. GAPDH was used as internal control. Results are presented as mean ± SEM (n=4). (C) Kinetics of STAT1 and phospho STAT1 activation in HuH7 cells transfected by nontagged *IFNL3* or *IFNL4* expressing plasmids detected by immunoblot analysis. ßActin was used as a loading control.

Supplementary Figure 2: **Densitometric quantification of STAT1 and pSTAT1.** Wild type HuH7 cells were transfected with non-tagged or Halo-tagged *IFNL3* or *IFNL4* for 48 hours or stimulated with recombinant IFNL3 or IFNL4 protein in the media for 24 hours. Empty plasmid was used as Mock control. Four independent experiments were used for quantification. **$p < 0.05$ was considered as statistically significant.

Supplementary Figure 3: **Characterization of IFNLR1 and IL10R2 knockout cell clones.** Confocal immunofluorescence images of IFNLR1 and IL10R2 were done in control and CRISPR/Cas9 edited HuH7 cells. Nuclei are shown in blue, membrane in green, IFNLR1 and IL10R2 are in red. Scale bar 20 µm. Representative pictures achieved from several knockout clones are displayed.

Supplementary Figure 4: **IFNLR1/IL10R2 receptor complex is indispensable for activation of JAK-STAT signaling pathway by IFNLs in HepG2 cells.** Control, IFNLR1 and IL10R2 knockout HepG2 cells were transfected with non-tagged *IFNL3* or *IFNL4* for 48 hours or stimulated with recombinant IFNL3 or IFNL4 for 24 hours. Empty plasmid was used as Mock control. Activation of STAT1 and pSTAT1 was determined by immunoblot analysis. Each lane is representative of 4 biological replicates per transfection/stimulation group. ßActin was used as a loading control.

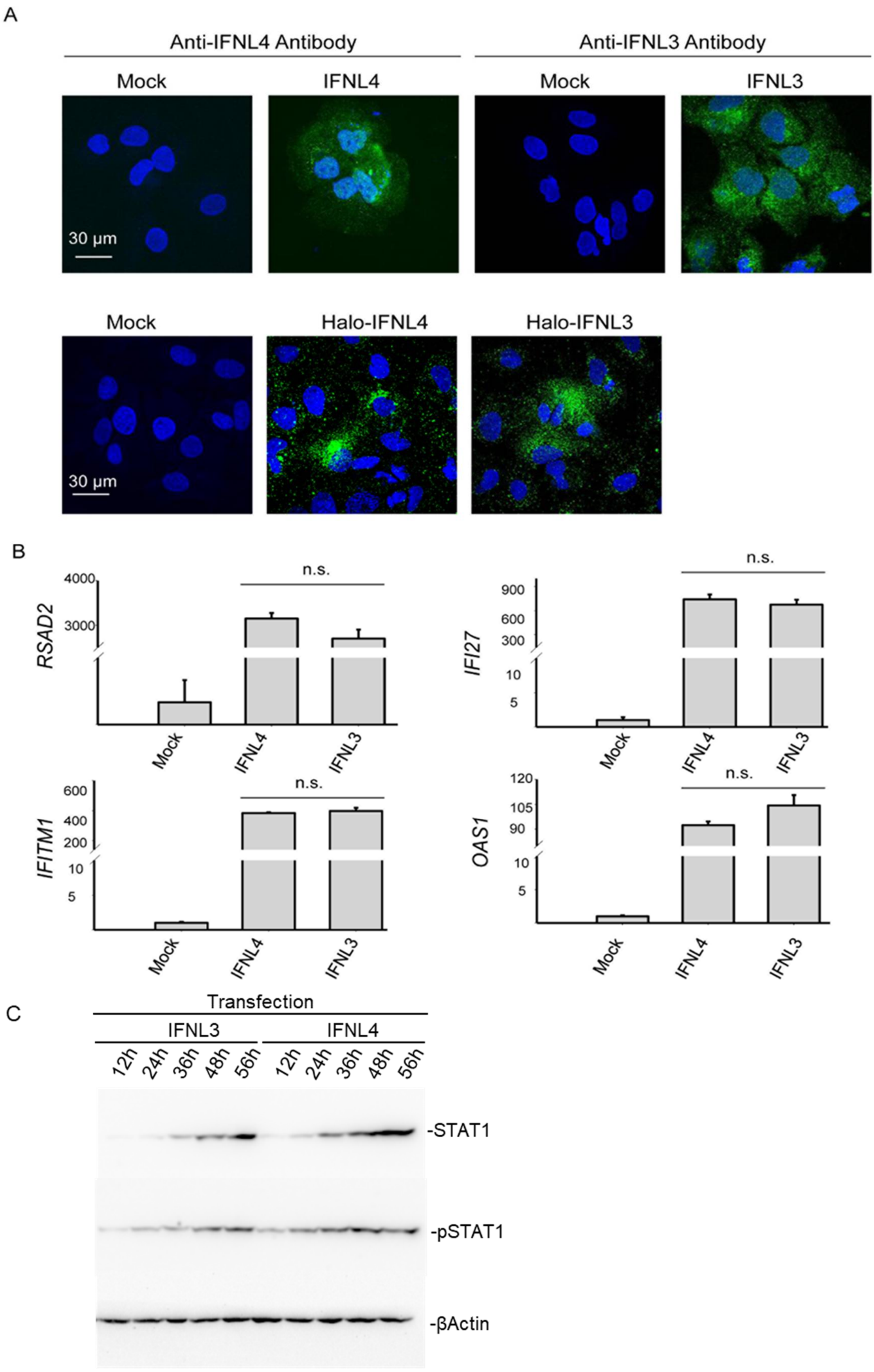

Supplementary Figure 1.

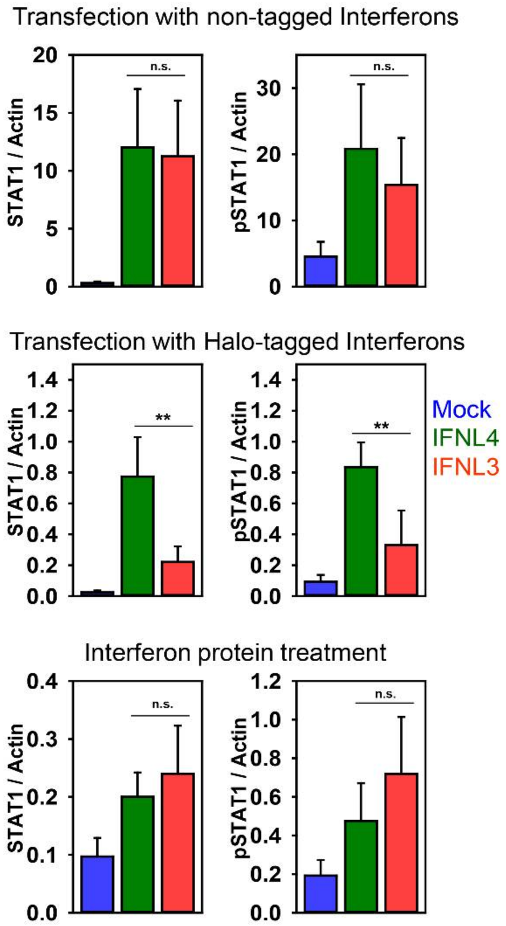

Supplementary Figure 2.

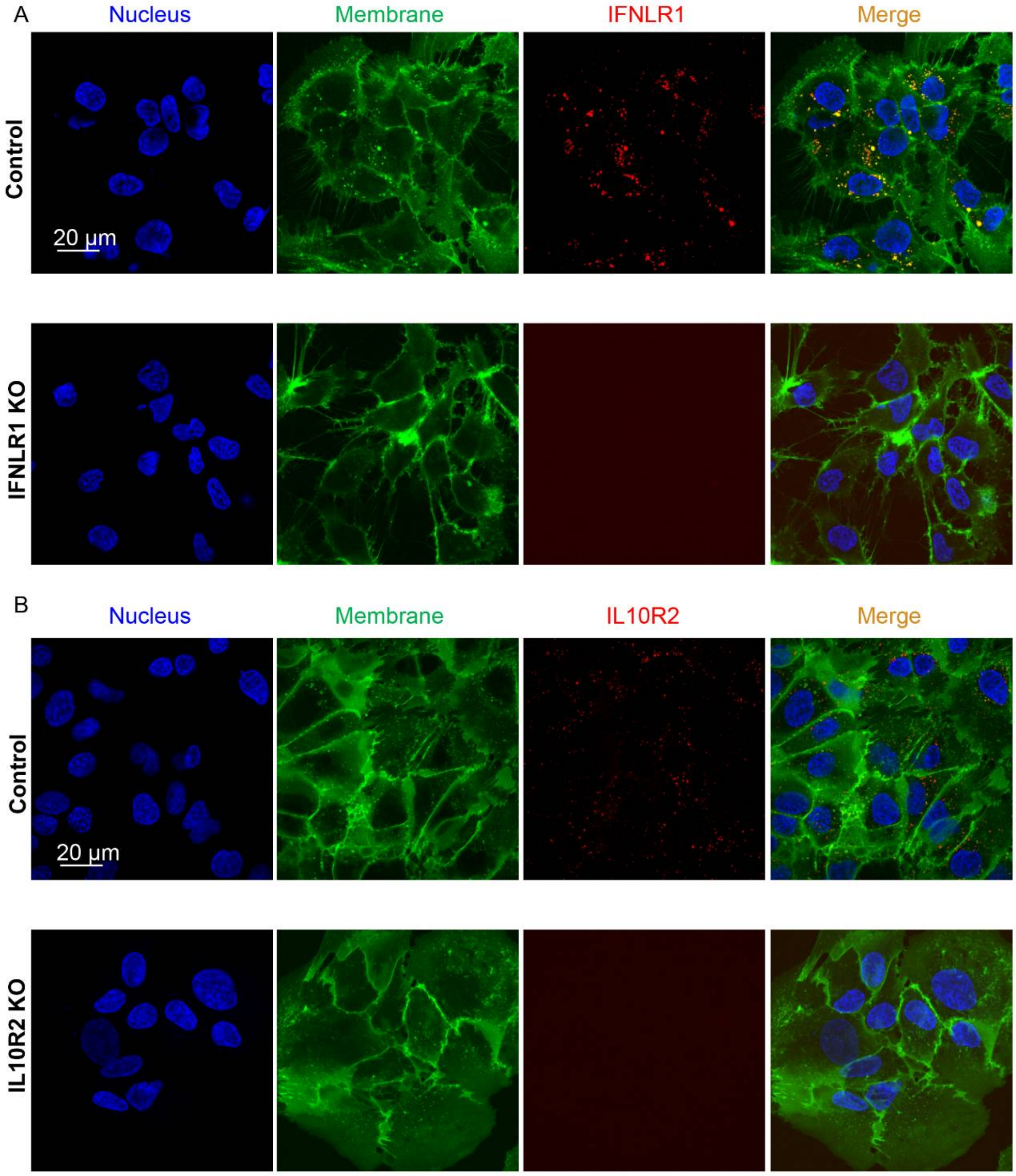

Supplementary Figure 3.

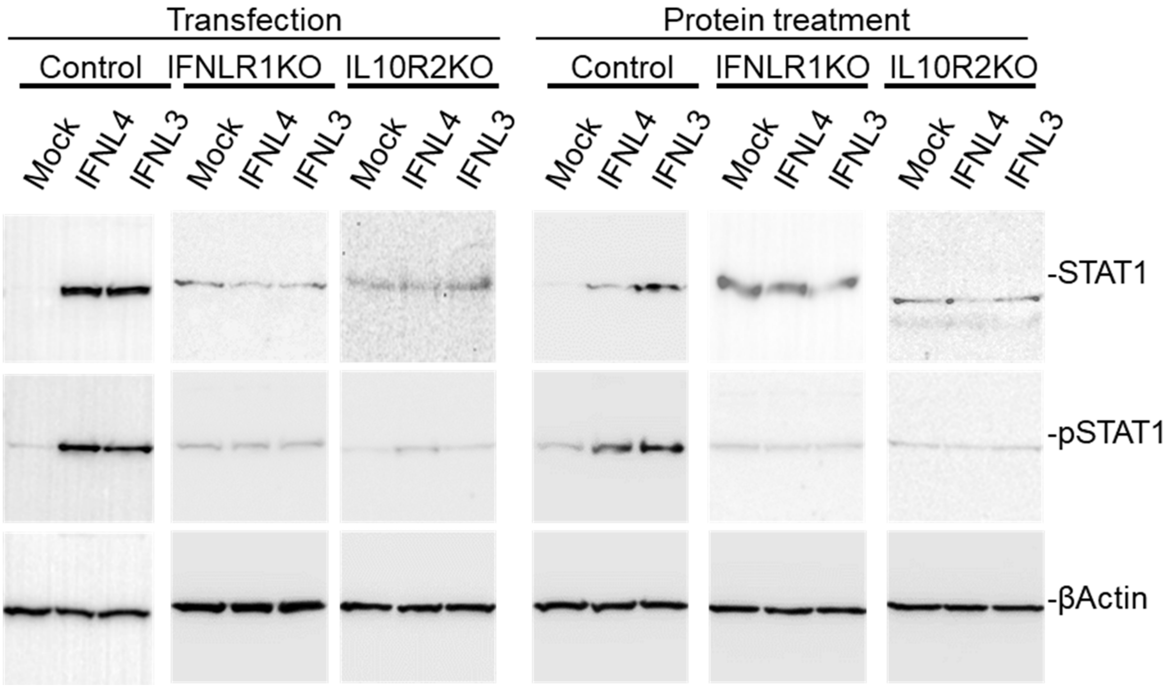

Supplementary Figure 4.

Supplementary Table 1: **Antibodies and chemicals used for immunoblotting and fluorescence labeling.**

**Name**	**Company**	**Cat №**	**Dilution**	
			**WB**	**IF**
βActin	ThermoFisher	MA5-15452	1:2000	
STAT1 (C-136)	Santa Cruz	SC-464	1:1000	
pSTAT1	Cell Signalling	8826	1:1000	
IFNL4	Millipore	MABF227		1:200
IFNL3	ThermoFisher	MA5-24254		1:200
IFNLR1	Novus Biologicals	NBP1-69636	1:500	
IL10R2	R&D Systems	MAB874-100	1:1000	
Goat anti mouse IgG	Invitrogen	G21040	1:10000	
Goat anti rabbit IgG	Invitrogen	G21234	1:10000	
Goat anti mouse Alexa 568/488	ThermoFisher	A-11004/A-11029		1:500
Goat anti rabbit Alexa 568/488	ThermoFisher	A-11011/A-11008		1:500
Cell Mask Green	ThermoFisher			1:1000
Hoechst	ThermoFisher	H1398		1:10000

Supplementary Table 2: **Description of IFNLR1 and IL10R2 knockout clones.**

Genotype	Protein	Clones
**IFNLR1**		
**HuH7**		
c.123delG	p.Thr42Hisfs*12	9x
c.125_126delCA	p.Thr42Metfs*24	4x
c.123_124insC	p.Thr42Hisfs*25	1x
**HepG2**		
c.122_126delTGACA	p.Thr42Alafs*23	2x
c.126_130delATGGC	p.Trp43Profs*22	1x
c.127_128delTG	p.Trp43Alafs*23	1x
c.121_125delCTGAC	p.Leu41Metfs*24	1x
**IL10R2**		
**HuH7**		
c.69delT	p.Glu24Lysfs*28	1x
c.59_60insA	p.Met20Ilefs*32	1x
**HepG2**		
c.22_23insT	p.Trp8Leufs*32	3x

Supplementary Table 3: **TaqMan gene expression assays used in real time PCR.**

Gene Name	TaqMan Gene Expression Assay ID
GAPDH	Hs02786624_g1
OAS1	Hs00973635_m1
MX1	Hs00895608_m1
ISG15	Hs00192713_m1
USP18	Hs00276441_m1
IFI6	Hs00242571_m1
IFI27	Hs00271467_m1
IFITM1	Hs01652522_g1
RSAD2	Hs00369813_m1
